# Comparison of Sexual Risk, HIV/STI Prevalence and Intervention Exposure Among Men Who Have Sex with Men and Women (MSMW) and Men Who Have Sex with Men Only (MSMO) in India: Implications for HIV Prevention

**DOI:** 10.1007/s10461-015-1058-2

**Published:** 2015-04-17

**Authors:** Lakshmi Ramakrishnan, Shreena Ramanathan, Venkatesan Chakrapani, Prabuddhagopal Goswami, Sucheta Deshpande, Diwakar Yadav, Shrabanti Sen, Bitra George, Ramesh Paranjape

**Affiliations:** No 11 Avatar Apartments, 27 Balakrishnan Road, Valmikingar, Thiruvanmiyur, Chennai, 600041 India; FHI 360 India, New Delhi, India; Centre for Sexuality and Health Research and Policy (C-SHaRP), Chennai, India; National AIDS Research Institute, Pune, Maharashtra India

**Keywords:** Bisexual behavior, Men who have sex with men, Men who have sex with men and women, India, HIV, Condom use, Bisexual concurrency

## Abstract

Using data from a cross-sectional bio-behavioral survey conducted among men who have sex with men (*n* = 3833) in India, we examined differences related to HIV-related sexual risk, HIV/STI prevalence and intervention exposures between men who have sex with men and women (MSMW, 35 % of the sample) and men who have sex with men only (MSMO). Among MSMW, 93 % reported having female regular partners, 14 % had female paid partners, and all types of male partners (regular 55 %; casual 77.1 %; paying 47 %; paid 19 %). Logistic regression revealed that MSMW had higher odds of being aged 26 years and above (AOR 4.45, 95 % CI 3.66–5.42), lower odds of inconsistently using condoms with male partners (AOR 0.82, 95 % CI 0.67–0.98) and lower odds of having kothi (feminine/mostly receptive) identity (AOR 0.07, 95 % CI 0.06–0.09). HIV intervention exposure and HIV/STI prevalence did not differ significantly between MSMW and MSMO (HIV 13.1 vs. 12.2 %; active syphilis 3.5 vs. 3.1 %, respectively). Concurrent sexual partnerships with men and women pose risk of HIV transmission/acquisition for MSM and their male and female partners. All subgroups of MSM require tailored information and skills to consistently use condoms with different types of partners of either gender.

## Introduction

Globally, men who have sex with men (MSM) have significantly high HIV prevalence, due to structural and individual level vulnerabilities [1]. In India too, MSM are at a higher risk of HIV infection, with a national average HIV prevalence of 4.4 % compared to 0.27 % among the general population [2]. The term ‘men who have sex with men’, in general, refers to any men who have sex with other men, regardless of their sexual orientation or sexual identity, whether or not they also have sex with women. However, for the purpose of this paper, the terms ‘men who have sex with men only’ (MSMO) and ‘men who have sex with men and women’ (MSMW) are used because the differences between these two groups may offer insights into differential sexual risks and HIV prevention targeting.

Among MSM from lower socioeconomic class, across India, certain identities such as kothi, double-decker and panthi are more common than gay or bisexual identities. Kothi refers to those same-sex attracted males who are feminine and primarily adapt receptive sexual role, double-decker refers to those who adapt insertive or receptive sexual role, and panthi refers to masculine men who primarily adapt insertive sexual role [3, 4]. There are minor regional variations in this terminology. For example, instead of ‘panthi’, the terms ‘parikh’ or ‘giriya’ are used in certain parts of North India, and instead of ‘double-decker’, the terms ‘do-paratha’ or ‘dupli’ are used in western and eastern parts of India [5]. Gay and bisexual identities are more common among same-sex or both-sex attracted males, respectively, from educated and middle or upper socioeconomic class.

A significant proportion of same-sex attracted men in India, irrespective of their sexual identities, are married or eventually get married due to societal expectations and family pressure, as getting married is seen as a duty to one’s family and to sustain one’s lineage [6, 7]. For example, the first Integrated Behavioral and Biological Assessment (IBBA) study documented that about one-fourth of the MSM participants had ever been married to women (16–37 %)—with 11.2 % of kothis, 25 % of double-deckers, 20 % of panthis, and 61.2 % of bisexual-identified MSM reported having ever been married [8]. Thus, bisexual behavior is not seen only among men who identify as ‘bisexual’, but also among MSM with diverse self-identities. Despite this awareness of the extent of bisexual behavior among even self-identified MSM, in general, HIV prevention interventions among MSM in India have remained predominantly focused on sexual risk reduction with male partners, with very limited attention to risk behaviors with female partners. Only recently the national HIV program explicitly acknowledged the need to address bisexual behavior among self-identified MSM and to promote safer sex with female partners of MSM as well [9]. Very few HIV programs focus on female partners of MSM in India. For instance, the Humsafar Trust, a community-based agency working with MSM in Mumbai, refers the female partners of its MSM clients to Family Planning Association of India (FPAI), where female partners are provided voluntary counselling and testing for sexually transmitted infections and HIV.

A recent meta-analysis on studies among MSMW in USA found that MSMW have relatively lower HIV prevalence when compared with MSMO, and less likely to engage in unprotected anal sex [10]. In India, there is limited and conflicting data on the possible differences, if any, on sexual risk behaviors and other factors associated with the prevalence of HIV and other sexually transmitted infections (STIs) among MSMW and MSMO. This means little understanding about whether and in what ways HIV prevention interventions need to be tailored depending on whether a person is MSMO or MSMW. One study from Bengaluru city in India that explicitly examined differences between MSMW and MSMO reported that MSMW were less likely to practice unprotected anal sex when compared with MSMO [11]. However, it did not examine differences in STI/HIV prevalence or intervention exposure.

Among the studies that reported HIV prevalence among married and unmarried MSM, one clinic-based study from Mumbai [12] and a multi-site community-based study [13] reported that married MSM were more likely than unmarried MSM to be HIV-positive, but another clinic-based study from Mumbai [14] could not find a statistically significant difference in HIV prevalence between married and single MSM.

To address these gaps in information and in order to inform designing HIV interventions tailored to MSMW’s prevention needs, the aim of this paper is to compare and contrast HIV-related sexual risk behaviors, prevalence of HIV/STIs and HIV intervention exposure among MSMW and MSMO.

## Methods

### Data Sources

Data from a cross sectional bio-behavioral survey conducted among MSM in 2009/2010 were used for this analysis. The survey was conducted in 10 districts of three southern states of India—Andhra Pradesh, Maharashtra and Tamil Nadu. The inclusion criteria slightly differed in these states. In Andhra Pradesh and Maharashtra, the inclusion criterion was ‘males aged 18 years and above who had any type of sex (oral, anal, or manual) with other males in the past one month’, whereas in Tamil Nadu the inclusion criterion was ‘males aged 18 years and above who had anal sex with other males in the past month in exchange for cash or in kind’. Following a rigorous sampling frame development, MSM were randomly sampled using time-location cluster sampling from cruising sites such as bus stands, cinema halls, parks, public toilets and other public places where MSM meet their potential male sexual partners. Two-stage sampling method was employed: at first stage, time-location clusters were selected using probability proportional to size (PPS); and in the second stage, respondents were recruited randomly from the selected clusters [15]. Written informed consent was obtained, following which interviews were conducted in private setting and blood and urine samples were collected. Ethical approvals were provided by Protection of Human Subjects Committee (PHSC) of FHI 360, Health Ministry Screening Committee (ICMR) and local ethical committees of the study implementing partners. Further details on the study methodology have been published elsewhere [16].

### Measures

#### Number and Types of Partners

Data were collected on the number and types of partners. Types of partners included: regular partner or main partner—a person with whom the respondent feels committed, such as spouse, lover or boyfriend/girlfriend; paying partner—a person who have paid the respondent cash or kind in exchange for sex; casual partner—a stranger, friend or acquaintance with whom the respondent had sex and but not considered as a regular partner; and paid partner—a person with whom the respondent had paid money to have sex.

#### MSMW and MSMO

As mentioned earlier, two categories of MSM were created for this analysis: (a) MSMO: those who reported having male regular partner, those who had a male paying partner in the past week, those who had any male paid partner in past one month and those who reported having any male casual partner (such as lover), and not reporting sex with any female partner in the past month; (b) MSMW: included those who had any of these above male partners and those who also reported having a female regular partner (such as wife or lover) and female paid partner in the past month. Since timeframe for the different partners (male and female) were current or within the past month, it was considered to be concurrent partners and hereafter will be referred to as such.

#### Condom Use

Condom use with different types of male and female partners was considered as the main outcome variable indicative of sexual risk. The survey included questions on condom use at last sex and consistency of condom use in the recent past (no specific timeframe was provided) with each type (regular, casual, paid, and paying) of male or female partners. Last sex condom use was examined separately for each partner type among MSMO and MSMW.

The main dependent variable was self-reported inconsistent condom use with any one of the four types of partners—separately examined for male and female partners. For each type of partner, participants who reported using condoms every time (during anal or vaginal sex) were considered to be consistent condom users, and those who reported using condoms most of the time, sometimes, and never were considered as inconsistent condom users. ‘Inconsistent condom use with any type of male partners’ was defined as having reported inconsistent condom use with any one of the four types (regular, casual, paying or paid) of male partners. Similarly ‘inconsistent condom use with any type of female partners’ was defined as having reported inconsistent condom use with any one of the two types (regular or paid) of female partners.

#### HIV and STIs

Blood samples were tested for HIV infection with a two-test algorithm using an enzyme immunoassay (J. Mitra, New Delhi) [16]. Blood samples were also tested for syphilis using rapid plasma reagin (RPR) and a confirmatory *Treponema pallidum* hemagglutination assay (TPHA). Positive RPR confirmation by TPHA was used to define reactive syphilis or lifetime syphilis. RPR titres of ≥1:8 with a confirmatory TPHA were defined as active or high-titre syphilis. Urine was tested using nucleic-acid amplification (Gen-Probe APTIMA COMBO 2) to assess Chlamydia (CT) and gonococcal (GC) urethral infections [16]. For the analysis, a composite variable ‘any STI’ was defined as those testing positive for either syphilis, NG or CT.

#### Socio-demographic and HIV Intervention Exposure Variables

These include age, literacy status (reading and writing), years of education, marital status, occupation and sexual self-identification. The latter included: kothis—predominantly receptive partner during penetrative sex with men; double-deckers (DD)—both insertive and receptive; and panthis—predominantly insertive [17, 18]. Alcohol use was also asked, with frequent alcohol use defined as those who consumed alcohol every day or at least once a week [19]. Other measures included were: HIV self-risk perception (yes or no) and having ever taken an HIV test and collected the result (yes or no). Exposures to HIV intervention services were examined: contacted by peer educators for HIV information, received condoms from HIV intervention staff, and visited program STI clinic in the past year. In addition, membership in community-based organization of MSM (a formal group comprised of and managed by MSM) was also included.

## Data Analysis

Data from all districts of the three states were merged for analysis. All estimates presented are not weighted. Bivariate and multivariable analyses were conducted using Stata (version 11.0). Chi square test was used to assess the associations between the independent variables and condom use outcome measures described above, with each of the partner type. Logistic regression was also conducted to identify differences, between MSMW and MSMO in terms of sexual risk behaviors (consistent condom use; condom use in last sex) and HIV/STI prevalence. Multivariable logistic regression was conducted: (a) to assess correlates of MSMW; and (b) to assess the sexual risk among MSMW and MSMO (for inconsistent condom use with any type of partners—separately for male and female partners). Odds ratios and 95 % confidence intervals (CI) were calculated after controlling for background variables in each model. Associations were considered significant for *p* value less than 0.05.

## Results

### Sample Characteristics

Among the total sample of 3739 MSM, about one-third (35 %, *n* = 1343) were classified as MSMW and remaining (65 %, *n* = 2396) as MSMO. Three-fourths (75 %) of MSMO self-identified as kothi, whereas nearly half (46 %) of MSMW identified as bisexual (*p* < 0.01). When compared with MSMW, significantly higher proportions of MSMO were young (18–25 years), completed secondary education, had never been married and reported sexual debut before 16 years of age. Almost equal proportion of MSMW and MSMO (40 and 38 %, respectively) reported frequent use of alcohol (every day or once a week) (Table 1).

**Table 1:** Profile of men have sex with both men and women (MSMW) and men who have sex with men only (MSMO), IBBA-2, 2009/2010

Variables	“MSMW” (*n* = 1343) % (*n*)	“MSMO” (*n* = 2396) % (*n*)	*p* value
Socio-demographics			
Self-identity			
Kothi	23.3 (314)	74.5 (1785)	0.000
Panthi	15.7 (211)	12.4 (298)	
Double-decker	14.7 (198)	10.1 (243)	
Bisexual	46.1 (620)	2.9 (70)	
Age group (years)			
18–25	29.4 (395)	57.1 (1369)	0.000
26–35	47.3 (636)	34.3 (825)	
36 and older	23.2 (312)	8.5 (204)	
Education			
Illiterate	18.0 (243)	9.6 (230)	0.000
Up to secondary education	32.3 (435)	27.5 (661)	
Above secondary education	49.5 (665)	62.8 (1505)	
Current marital status			
Never married	24.0 (323)	95.9 (2298)	0.000
Currently married	75.4 (1013)	2.6 (64)	
Divorced/widowed/separated	0.52 (7)	1.4 (34)	
Occupation			
Unemployed/students	7.1 (96)	14.9 (357)	0.000
Manual laborers	31.9 (429)	28.5 (683)	
Business/Govt. or Pvt. employee/professional	58.3 (783)	49.7 (1192)	
Masseur/sex worker	1.7 (23)	6.1 (148)	
Transport workers	0.8 (12)	0.6 (16)	
Age at sexual debut with a male			
Less than 16	23.4 (315)	44.0 (1055)	0.000
16 and above	76.5 (1028)	55.9 (1341)	
Age at sexual debut with a female			
Less than 16	2.2 (30)	6.2 (19)	0.000
16 and above	97.7 (1280)	93.7 (286)	
Alcohol use			
Everyday	5.3 (72)	6.0 (144)	0.002
Once a week	35.0 (471)	32.0 (767)	
Less than once a week	22.2 (299)	19.1 (458)	
Not in the past month	6.8 (92)	6.4 (154)	
Never	30.4 (409)	36.4 (873)	
Risk perception and HIV testing			
Self-perceived HIV risk			
No	79.2 (1064)	73.2 (1755)	0.000
Yes	20.7 (279)	26.7 (641)	
Ever been tested and returned to collect HIV test results			
No	24.9 (335)	18.6 (446)	0.000
Yes	75.0 (1008)	81.3 (1950)	
Use of HIV-related prevention services (in the past year)			
Received peer education			
No	30.5 (410)	20.4 (490)	0.000
Yes	69.4 (933)	79.5 (1906)	
Received condoms from NGOs/CBOs			
No	31.0 (417)	20.3 (488)	0.000
Yes	68.9 (926)	79.6 (1908)	
Visited NGO-managed STI clinics			
No	34.2 (460)	25.8 (620)	0.000
Yes	65.7 (883)	74.1 (1776)	
Composite indicator: exposure to HIV intervention			
No	29.5 (397)	19.4 (465)	0.000
Yes	70.4 (946)	80.5 (1931)	
Membership in a CBO			
No	50.3 (676)	46.5 (1115)	0.026
Yes	49.6 (667)	53.4 (1281)	
HIV and STIs (lab tests)			
HIV			
Negative	86.9 (1167)	87.7 (2103)	0.438
Positive	13.1 (176)	12.2 (293)	
Syphilis			
Negative	92.9 (1248)	94.4 (2263)	0.062
Positive	7.0 (95)	5.5 (133)	
High-titre syphilis			
Negative	96.5 (1296)	96.8 (2320)	0.590
Positive	3.5 (47)	3.1 (76)	
Any STI (excluding HIV)			
Negative	92.2 (1239)	93.6 (2243)	0.115
Positive	7.7 (104)	6.3 (153)	

*NGO/CBO* Non-governmental Organization/Community-Based Organization

## HIV-Related Risk Perception, Exposure to HIV Intervention and STI/HIV Prevalence

### Overview of MSMW

Although a majority of MSMW did not perceive to be at risk of HIV, in the past year, 75 % had tested for HIV and returned to collect test results. About two-thirds (70 %) of MSMW had received services for HIV prevention (peer education, free condoms, and STI clinic check-ups) and 50 % reported being members of community-based organizations. Thirteen percent tested positive for HIV and 3.5 % had evidence of active syphilis.

### Comparison of MSMW to MSMO

A relatively lower proportion of MSMW perceived themselves at risk for HIV (21 vs. 27 %, *p* < 0.001), and had ever been tested for HIV and collected test results (75 vs. 81 %, *p* < 0.001) compared to MSMO. Exposures to any type of HIV intervention services as well as CBO membership were significantly lower among MSMW. No significant differences were observed in HIV or STI prevalence among two groups (Table 1).

## Partner Characteristics and Sexual Behaviors

### Overview of MSMW

Over 75 % of MSMW were married and living with a female partner. However, only 4 % of MSMW reported that their female regular partners were aware of their same-sex sexual behavior. MSMW reported having all types of male partners concurrently within the past month in the following order: male casual partners (77 %), male regular partners (55 %) and paying partners (47 %). A vast majority (93 %) reported having a current female regular partner and 14.6 % reported having sex with a female sex worker in the past month (Table 2).

**Table 2:** Partners-related characteristics and sexual risk behaviors of MSMW and MSMO

Variables	“MSMW” (*n* = 1343) % (*n*)	“MSMO” (*n* = 2396) % (*n*)	*p* value
Types of partners^a^			
Male regular partners			
No	44.2 (594)	25.7 (616)	0.000
Yes	55.7 (749)	74.2 (1778)	
Male paying partners			
No	52.9 (711)	27.3 (656)	0.000
Yes	47.0 (632)	72.6 (1740)	
Male paid partners			
No	80.9 (1086)	86.6 (2074)	0.000
Yes	19.0 (256)	13.3 (319)	
Male casual partners			
No	22.8 (307)	29.6 (709)	0.000
Yes	77.1 (1035)	70.4 (1686)	
Female regular partners			
No	6.2 (84)	–	0.000
Yes	93.7 (1259)	–	
Female paid partners			
No	85.3 (1146)	–	0.000
Yes	14.6 (197)	–	
Characteristics of female regular and paid partners of MSMW			
Currently married			
No	24.5 (330)	–	–
Yes	75.4 (1013)	–	–
*Regular female partner*			
Living together			
No	23.2 (312)	–	–
Yes	76.7 (1031)	–	–
Female regular partner’s awareness of husband’s same-sex sexual behavior			
No	96.2 (1292)	–	–
Yes	3.8 (51)	–	–
Number of sex acts with female regular partner in the past month			
≤7	52.7 (617)	–	–
8–14	16.3 (191)	–	–
15 and above	30.8 (361)	–	–
*Paid partner*			
Number of times bought sex with females in the past one month			
≤4	82.6 (148)	–	–
5 and above	17.3 (31)	–	–
Condom use behaviors			
*Last time condom use (anal or vaginal sex)*			
With male regular partner			
No	10.6 (76)	13.3 (229)	0.071
Yes	89.3 (637)	86.6 (1490)	
With male paying partner			
No	2.2 (14)	1.8 (30)	0.455
Yes	97.7 (598)	98.2 (1635)	
With male paid partner			
No	6.3 (16)	6.0 (19)	0.910
Yes	93.7 (238)	93.9 (294))	
With male casual partner			
No	2.0 (20)	1.6 (26)	0.466
Yes	97.9 (944)	98.3 (1527)	
With female regular partner			
No	76.4 (955)	–	–
Yes	23.5 (294)	–	–
With female paid partner			
No	4.5 (9)	–	–
Yes	95.4 (188)	–	–
*Consistent condom use (in general)*			
With regular male partners			
No	27.5 (187)	34.2 (564)	0.002
Yes	72.4 (492)	65.8 (1085)	
With male paying partners			
No	29.1 (178)	31.4 (525)	0.284
Yes	70.8 (433)	68.5 (1143)	
With male paid partners			
No	18.6 (47)	22.9 (71)	0.211
Yes	81.3 (205)	77.0 (238)	
With casual male partners			
No	16.8 (163)	22.2 (349)	0.001
Yes	83.1 (804)	77.7 (1222)	
With regular female partners			
No	85.3 (1067)	–	–
Yes	14.6 (183)	–	–
With paid female partners			
No	17.2 (34)	–	–
Yes	82.7 (163)	–	–
Inconsistency in condom use with			
Any type of male partners (regular, paying, paid, or casual)	27.0 (363)	35.2 (844)	0.000
Female partners (paid or regular)	77.0 (1035)	–	0.000
Both male and female partners	23.8 (320)	–	0.000
Lubricant use^a^			
Non-users/mixed users	74.2 (995)	60.7 (1453)	0.000
Exclusive use of water-based lubricants	25.8 (346)	39.2 (937)	

Total and % may not add up due to missing values

^a^Mixed users include those who reported using both water and oil based lubricants

The percentage of condom use during last sexual act reported by MSMW was high with all types of male partners: with male regular partners it was 89 % and with other male partners it was over 90 %. Consistent condom use among MSMW was higher among casual and paid male partners (over 80 %) compared with male regular (72.4 %) and paying partners (70.8 %).

Last sex condom use was higher with female paid partners (93 %) (23 %). Similarly, consistent condom use was only 14.6 % with female regular partners, but higher with female paid partners (83%).

### Comparison of MSMW to MSMO

About 3 % of MSMO reported being currently married. Similar to MSMW, MSMO reported having all types of male partners. However, MSMO had higher proportion of male regular partners (74 %) and male paying partners (73 %), but fewer male paid and male casual partners. There was no statistically significant difference between MSMW and MSMO in condom use during last anal sex with any type of male partner (Table 2).

However, when compared with MSMW, consistent condom use was lower among all types of male partners of MSMO. With significantly lower proportion of MSMO reporting consistent condom use with male regular (65.8 vs. 72.4 %, *p* < 0.01) and with male casual (77.7 vs. 83.1 %, *p* < 0.01) partners (Tables 3, 4).

**Table 3:** Multivariate associations of being men who have sex with men and women—MSMW (vs. MSMO), IBBA-2, 2009/2010 (*n* = 1343)

Variables	MSMW	*p* value
	Adjusted odds ratio (95 % confidence interval)	
Self-identity		
Non-kothi^a^	Referent	
Kothi	0.07 (0.06–0.09)	0.000
Age (years)		
≤25	Referent	
26 and older	4.45 (3.66–5.42)	0.000
Education		
Illiterate	Referent	
Literate	0.44 (0.35–0.55)	0.000
Occupation		
Organized sector (Govt./Private), unemployed/students	Referent	
Manual laborers	2.18 (1.64–2.91)	0.000
Alcohol use		
No	Referent	
Yes	1.11 (0.94–1.33)	0.218
Having male regular partner		
No	Referent	
Yes	0.75 (0.62–0.89)	0.001
Having male paying partners		
No	Referent	
Yes	0.91 (0.75–1.12)	0.400
Having male casual partners		
No	Referent	
Yes	0.94 (0.77–1.15)	0.532
Inconsistent condom use with any type of male partners (regular, paying, paid, casual)		
No	Referent	
Yes	0.82 (0.67-0.98)	0.036
Composite indicator: exposure to HIV intervention		
No	Referent	
Yes	1.19 (0.96–1.47)	0.104
Self-perceived HIV risk		
No	Referent	
Yes	0.93 (0.76–1.14)	0.473
Membership in a CBO		
No	Referent	
Yes	0.99 (0.99–1.00)	0.909
Exclusive use of water-based lubricants		
No	Referent	
Yes	0.79 (0.66–0.96)	0.019
Syphilis		
Negative	Referent	
Positive	1.10 (0.78–1.55)	0.577

^a^‘Non-kothi’ includes MSM who identified as panthis, double-deckers, or bisexuals

Model *χ* ^2^ (16) = 1439.45, *p* < 0.001, log likelihood = −1711.78, pseudo *R* ^2^ = 0.296

**Table 4:** Multivariate associations of inconsistent condom use with any type (regular, casual, paying or paid) of male partners and female partners (regular or paid) among MSMW and MSMO, 2009/2010

Variables	MSMW				MSMO	
	Inconsistent condom use with any type of male partners^a^		Inconsistent condom use with any type of female partners^b^		Inconsistent condom use with any type of male partners^c^	
	Adjusted odds ratio (95 % CI)	*p* value	Adjusted odds ratio (95 % CI)	*p* value	Adjusted odds ratio (95 % CI)	*p* value
Self-identity						
Kothi	Referent		Referent		Referent	
Panthi	0.51 (0.31–0.85)	0.009	0.15 (0.07–0.31)	0.000	0.35 (0.25–0.50)	0.000
Double-decker	0.87 (0.59–1.28)	0.487	0.36 (0.18–0.75)	0.006	0.71 (0.53–0.96)	0.028
Bisexual	0.40 (0.28–0.56)	0.000	0.29 (0.16–0.54)	0.000	0.36 (0.20–0.64)	0.001
Age (years)						
≤25	Referent		Referent		Referent	
26 or older	1.62 (1.07–2.46)	0.022	6.8 (4.84–9.66)	0.000	1.59 (1.32–1.91)	0.000
Education						
Illiterate	Referent		Referent		Referent	
Literate	1.02 (0.75–1.38)	0.885	0.42 (0.262–0.66)	0.000	1.02 (0.77–1.34)	0.863
Currently married						
No	Referent		–		–	
Yes	0.79 (0.52–1.18)	0.253	–	–	–	–
Occupation						
Organized sector/unemployed/students	Referent		Referent		Referent	
Manual laborers	1.42 (0.78–2.56)	0.240	1.54 (0.91–2.61)	0.108	1.04 (0.80–1.37)	0.729
Alcohol use						
No	Referent		Referent		Referent	
Yes	1.67 (1.27–2.19)	0.000	0.67 (0.48–0.93)	0.017	1.48 (1.24–1.78)	0.000
Received peer education						
No	Referent		Referent		Referent	
Yes	1.98 (0.77–5.07)	0.155	1.52 (0.55–4.18)	0.417	0.64 (0.30–1.36)	0.252
Received condoms from NGOs/CBOs						
No	Referent		Referent		Referent	
Yes	0.93 (0.37–2.29)	0.870	1.00 (0.39–2.50)	0.983	0.93 (0.44–1.36)	0.245
Visited NGO-managed STI clinics						
No	Referent		Referent		Referent	
Yes	0.57 (0.21–0.66)	0.001	1.10 (0.54–2.29)	0.780	0.77 (0.54–1.09)	0.151
Self-perceived HIV risk						
No	Referent		Referent		Referent	
Yes	1.58 (1.17–2.13)	0.002	0.77 (0.51–1.14)	0.196	1.14 (0.94–1.38)	0.198
Female regular partner’s awareness of husband’s same-sex sexual behavior						
No	NA		Referent		NA	–
Yes			0.36 (0.16–0.81)	0.015	–	
Membership in a CBO						
No	Referent		Referent		Referent	
Yes	0.99 (0.98–1.00)	0.588	1.00 (0.99–1.01)	0.324	0.99 (0.98–1.00)	0.381
Exclusive use of water-based lubricants						
No	Referent		–		Referent	
Yes	1.05 (0.78–1.42)	0.736	–	–	1.08 (0.91–1.31)	0.364
Syphilis						
No	Referent		Referent		Referent	
Yes	0.96 (0.58–1.58)	0.859	0.88 (0.45–1.76)	0.736	1.58 (1.07–2.32)	0.021
HIV						
Negative	Referent		Referent		Referent	
Positive	0.81 (0.55–1.19)	0.285	1.42 (0.81–2.50)	0.219	0.75 (0.56–0.99)	0.043
Inconsistent condom use with any type of male partners						
No	NA		Referent		–	
Yes	–	–	7.45 (4.9–12.34)	0.000	–	–

^a^Prob > *χ* ^2^ = 0.000, log likelihood = −88.5, pseudo *R* ^2^ = 0.057

^b^Prob > *χ* ^2^ = 0.000; log likelihood = −445.52, pseudo *R* ^2^ = 0.308

^c^Prob > *χ* ^2^ = 0.000; log likelihood = −138.04, pseudo *R* ^2^ = 0.045

When compared with MSMW, higher proportion of MSMO (35.2 vs. 27 %, *p* < 0.001) reported inconsistent condom use with any type of male partners. Similarly, use of exclusive water-based lubricants was higher among MSMO (39 vs. 26 %, *p* < 0.001).

### Findings from Multivariate Analysis

#### Overview of MSMW

Multivariate analysis identified significant correlates of MSMW group. MSMW were less likely to identify as kothi [AOR 0.07 (0.05–0.09)] and more likely to be >26 years of age [AOR 4.49 (3.69–5.47)]. MSMW were less likely to have a male regular partner [AOR 0.73 (0.60–0.87)] compared to MSMO; however, there was no significant differences from MSMO in having other types of male partners. MSMW were less likely to be inconsistent condom users with any type of male partners [AOR 0.73 (0.60–0.88)] compared with MSMO. While there was no difference in self-perception of HIV risk or exposure to services such as peer education and condoms, MSMW were more likely to have received STI clinical services [AOR 1.60 (1.10–2.32)] compared to MSMO.

## Associations with Inconsistent Condom Use: Comparison of MSMW and MSMO

### Inconsistent Condom Use with Male Partners

Correlates of inconsistent condom use with any type of male partners was examined separately for MSMW and MSMO. The common factors that were found significantly associated with inconsistent condom use with any type of male partners were: age >26 years [MSMW: AOR 1.62 (1.07–2.46); MSMO: AOR 1.58 (1.32–1.90)] and frequent alcohol use [MSMW: AOR 1.67 (1.28–2.19); MSMO: AOR 1.48 (1.24–1.78)].

#### Significant Unique Correlates in MSMW Group

Among MSMW, with kothis as the reference group, inconsistent condom use with any type of male partners was less likely among panthis [AOR 0.51 (0.30–0.85)] and bisexual-identified MSM [AOR 0.40 (0.28–0.56)]. Among MSMO, with kothis as the reference group, inconsistent condom use with any type of male partners was significantly lower among all other subgroups [panthis = AOR 0.35 (0.25–0.50); bisexuals = AOR 0.36 (0.20–0.64); DDs = AOR 0.35 (0.20–0.65)].

Among MSMW, those who were exposed to STI clinical services were less likely to be inconsistent condom users with any type of male partners [AOR 0.57 (0.21–0.66)]. Other two HIV intervention exposure variables (having received peer education or condoms) were not found to have statistically significant association with inconsistent condom use with both MSMW and MSMO groups. Presence of self-perceived HIV risk was found to be significantly associated with inconsistent condom use with any type of male partners among MSMW [AOR 1.44 (1.06–1.94)], but not with MSMO.

#### Significant Unique Correlates in MSMO Group

MSMO who were syphilis positive [AOR 1.58 (1.07–2.32)] were more likely to be inconsistent condom users; whereas, MSMO who were HIV positive [AOR 0.75 (0.56–0.99)] were less likely to be inconsistent condom users.

### Inconsistent Condom Use with Female Partners

Similar to the pattern observed in significant correlates of inconsistent condom use with male partners, MSMW who were 26 years and above [AOR 6.8 (4.84–9.66)] were more likely to be inconsistent condom users with any type of female partners. However, frequent alcohol users [AOR 0.42 (0.26–0.66)] were less likely to be inconsistent condom users with their female partners.

Similar to the pattern with male partners, compared to kothi-identified MSMW, MSMW who identified as panthi [AOR 0.15 (0.07–0.31)], DD [AOR 0.36 (0.18–0.75)] and bisexual [AOR 0.29 (0.16–0.54)] were significantly less likely to be inconsistent condom users with their female partners. Exposure to HIV intervention was not found to be associated with inconsistent condom use with female partners. MSMW who reported that their female partners were aware of their same-sex sexual behaviors [AOR 0.36 (0.16–0.81)] were found less likely to be inconsistent condom users with their female partners. MSMW who were inconsistent condom users with any type of male partners [AOR 7.45 (4.9–12.34)] were more likely to inconsistent condom users with any type of female partners as well.

## Discussion

This analysis based on a large cross-sectional survey among MSM in southern India has found that, when compared with MSMO, in general, MSMW have relatively less HIV-related sexual risk behaviors with their male partners, even after controlling for HIV intervention exposure. Despite this lower sexual risk among MSMW, there were no significant differences in HIV or STI prevalence between MSMW and MSMO. We found evidence that MSMW concurrently have unprotected sex with both male and female partners, increasing the chances of HIV transmission risk to partners of both sexes and to themselves.

### Prevalence of Bisexual Behavior

Our findings that about one-third of MSM engage in heterosexual behavior as well is consistent with findings from other published studies among MSM in India. For example, other studies conducted among community-based samples of MSM such as a study from Bengaluru [11] reported 30 % had bisexual behavior, and the first round of IBBA reported that 15–45 % of different subgroups of MSM had female regular partners [13].

### Differences in HIV and STI Prevalence Among MSMW and MSMO

The present study found that MSMW and MSMO had high prevalence of HIV and syphilis, similar in magnitude to the high HIV and STI prevalence found in a community-based [13] and a clinic-based study among MSM [12]. But unlike these two studies which reported that married MSM were more likely to have HIV infection than single MSM (29 vs. 18 % in Brahmam et al. [13] and 23 vs. 9 % in Kumta et al. [12]), the present study did not find any significant differences in HIV or syphilis prevalence among MSMW and MSMO, after controlling for HIV intervention exposure. It is possible that MSMW at higher risk might not have been captured in this study as after marriage many MSM stop coming to community-based agencies or even to cruising sites to avoid being discriminated by unmarried MSM [20].

### Age and Identity Differences Between MSMW and MSMO

We found that MSMW were more likely to be aged 26 years and above, and more likely to have non-kothi identities, consistent with the findings from another study that explicitly compared MSMW and MSMO in Bangalore city [11]. It has been previously documented that even same-sex attracted males in India eventually get married to woman as they see marriage as a duty to one’s family in this primarily collectivistic culture where family occupies a central role and arranged marriage is still widely prevalent [3, 21]. However, marital status was not a significant correlate of being MSMW, which means even in absence of marriage MSM in different subgroups have sex with females. The finding that MSMW are more likely to be non-kothi identities could be possibly explained by the relatively more fluid nature of sexual behaviors among other subgroups of MSM (panthis, double-deckers and bisexuals) when compared with kothis. However, it is also possible that in our sample, kothis might have concealed their bisexual behavior or their marital status because of the stigma within kothi communities in relation to bisexual behavior and marriage.

### Differences in Condom Use Between MSMW and MSMO

Our study findings showed that, when compared with MSMO, a higher proportion of MSMW consistently used condoms. Similarly, MSMW were less likely to be inconsistent condom users than MSMO with their male partners. These findings are consistent with the Bangalore study [11] that compared MSMW and MSMO, as well as a study that examined bisexual concurrency among South African MSM [22]. Our finding that MSMW, in general, have high rates of condom use than MSMO, could not be explained by exposure to HIV interventions, as it was not found to be significantly different between MSMW and MSMO.

Future qualitative studies can explore whether a sense of responsibility to prevent transmission of HIV to their wives and children motivates MSMW to be more consistent in condom use with their male partners. However, the proportions of MSMW with inconsistent condom use with their male partners of any type are still high enough to warrant further attention towards improving consistent condom use with their male partners.

### Condom Use with Female Partners

Being aged 26 years and above was significantly associated with inconsistent condom use with female partners whereas frequent use of alcohol was significantly less likely to be associated with inconsistent condom use with female partners. Also, when compared with kothis, MSMW who self-identified as panthi, DD or bisexual were less likely to be inconsistent condom users with their female partners, consistent with another study conducted among MSM in Bangalore, South India [6]. While the association between frequent alcohol use and inconsistent condom use can be understood [19], it is not clear how the self-identities of MSMW might account for the differences in their condom use with their female partners, given the paucity of research in this area in India.

Our key finding from bivariate analyses that those MSMW who reported inconsistent condom use with any one of the four types of male partners are also likely to be inconsistent condom users with their female partners indicate that partners of either gender of MSMW are at-risk for HIV and STIs, and MSMW too can get infected by or infect partners of either gender. Bivariate analyses also indicated that MSMW are more likely to inconsistently use condoms with their female regular partners compared to other types of female partners. This is possibly because condom use, in general, with in marital relationships is seen as affecting intimacy and possible indication of extra-marital sex [23]. Also, the widespread use of family planning technologies (oral contraceptives or tubectomy) by women in India may prevent married MSM from justifying the use of condoms as a contraceptive device [24].

Disclosure of one’s sexuality to spouse is very rare in our sample, as confirmed in other Indian studies [25, 26] and a South African study [22]. It is not clear whether disclosure of one’s sexuality can facilitate safer sex practices with their spouse, even though multivariate analysis in this study showed that those MSMW whose spouse were aware of their husband’s sexuality were more likely to report consistent condom use with their female partners. The connection between disclosure of one’s sexuality to spouse and other female partners, and condom use needs to be further examined through qualitative or mixed methods studies.

### Limitations

This current analysis has several limitations. The samples were from MSM accessing cruising sites in Western and Southern Indian states with a long history of HIV interventions among MSM. This issue, along with a minor difference in the inclusion criterion used in one of the three study states (Tamil Nadu), preclude generalizing the findings to even to MSM accessing cruising sites in India. Another limitation could be in relation to how we operationalized the definitions of MSMO and MSMW in this paper. While other studies from India [11] and abroad [27–29] have included a longer time-frame (1-year or ever) for having had sex with female to label a person as MSMW or MSMO, we used a relatively shorter timeframe of one month as we did not have data on whether the participants had sex with women in the previous 6 months or 1 year.

Another major limitation is the lack of explicit timeframe for measuring consistency of condom use, even though the responses were interpreted to be consistent condom use in the recent past. Similarly, timeframes for reporting the types of male and female partners were slightly different, although for practical considerations, sexual partnerships were considered as concurrent, if the partnership was mentioned as current (e.g., spouse or female regular partner) or within the past month, as the eligibility criteria was that the participant must have had sex with a man in the past month. As sensitive information about sexual behaviors and condom use were asked, social desirability might have affected the responses. While the association between frequency of alcohol use and condom use was examined in this paper, this study did not collect information on alcohol use before having sex. However, other studies among MSM in India [23, 24, 30] have reported that alcohol use before having anal sex was associated with lack of condom use. Where relevant, future quantitative studies on sexual risk (condom use) among MSM need to collect data on alcohol use before having sex.

### Implications

Like other studies from India, this study found bisexual behavior across all subgroups of MSM, including kothi-identified MSM. This means that assumptions regarding one’s sexual behavior or marital status should not be based on self-reported sexual identity alone. This has important implications for clinical and counselling practice in terms on asking the sexual history in a sensitive manner and providing appropriate safer sex information. A significant proportion of MSMW inconsistently use condoms with both male and female partners, therefore it is critical that HIV interventions specifically address the need to consistently use condoms with partners of either gender; and provide tailored support in using condoms with all types of female partners, especially female regular partners; and to encourage MSM in getting their female regular partners tested for HIV or STIs. Support also needs to be available to MSMW who wish to disclose their same-sex sexual behavior to female regular partners, as disclosure might possibly help in practicing safer-sex with female regular partners. Diversity in sexual behavior of self-identified MSM needs to be explicitly discussed with all subgroups of MSM and stigma associated with heterosexual marriage within the self-identified MSM communities needs be addressed so that MSM who are married or have female partners could then openly discuss the challenges they have in practicing safer sex with their female partners. Future research can specifically study bisexual concurrency with specified timeframes for different types of male and female partners and consistency in condom use, and also examine the prevalence of HIV and condom use in anal sex with different types of female partners. Bisexual behavior and bisexual concurrency (sex with partners of both genders, who may be regular, casual, paying or paid partners) among heterosexual-identified men too need to be studied to assess their HIV-related risk behaviors and to compare the differences between heterosexually-identified MSMW and non-heterosexually identified MSMW, using both quantitative and qualitative methods.

## Conclusion

Bisexual partnerships are commonly reported among diverse subgroups of self-identified MSM (such as kothis and double-deckers) in India. Our study found high HIV prevalence among both MSMW and MSMO, but it did not differ significantly between these two groups, after controlling for HIV intervention exposure. Also, MSMW, in general, were found to have high levels of inconsistent condom use with both male and female partners. HIV interventions among MSM need to acknowledge bisexual behavior among even self-identified MSM, and educate and counsel them on the risks associated with both unprotected anal and vaginal sex, and provide support for consistent use of condoms with partners of either gender. Married MSM also need to be trained on practical sexual communication and negotiation skills in dealing with the stigma surrounding condom use with spouse (which is seen as a sign of mistrust and infidelity). In addition, steps need to be taken to decrease the stigma faced by heterosexually-married self-identified MSM so that married MSM could access HIV-related services from community organizations as well as receive the necessary psychosocial support from their peers. Innovative ways of screening and treating female partners of self-identified MSM for HIV/STI, while maintaining the confidentiality of the sexuality of their husbands, need to be developed to decrease the risk of HIV/STI transmission and acquisition.
